# Supplementary figures and images for: Modeling the three-dimensional connectivity of in vitro cortical ensembles coupled to Micro-Electrode Arrays
Source: PLoS Comput Biol. 2023 Feb 13;19(2):e1010825. doi: 10.1371/journal.pcbi.1010825 (PMC9956882; doi:10.1371/journal.pcbi.1010825)

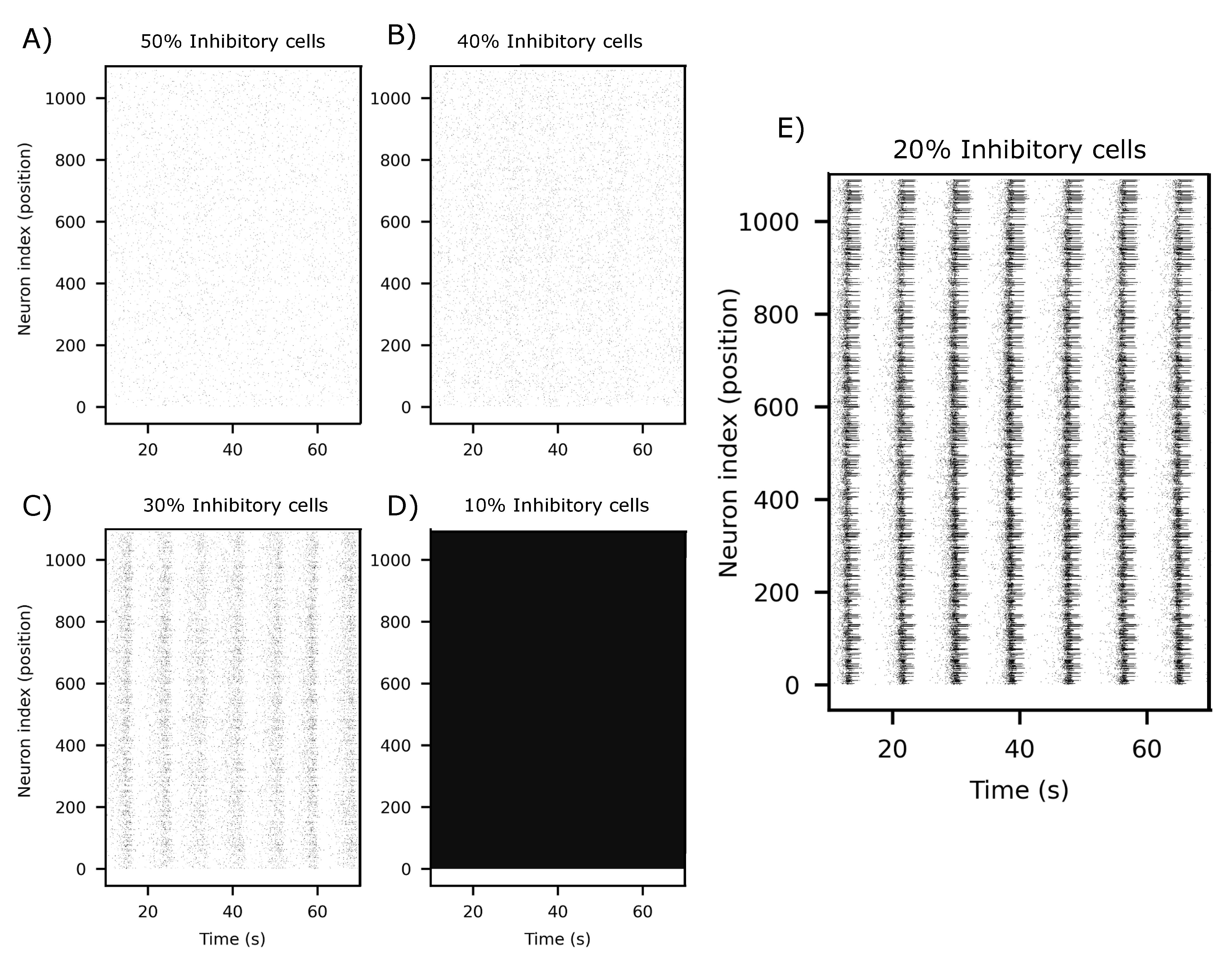

Supplement: S1 Fig — 60-s simulated spontaneous electrophysiological activity of representative 2D networks as a function of the percentage of inhibitory neurons. (A) 50%, (B) 40%, (C) 30%, (D) 10%, and (E) 20% of inhibitory neurons. This last configuration allows to reproduce values of firing and bursting rates comparable with the experimental recordings of mature 2D cortical cultures as well as the presence of population events (network bursts) involving most of the neurons of the network. (TIF) [file pcbi.1010825.s001.tif]

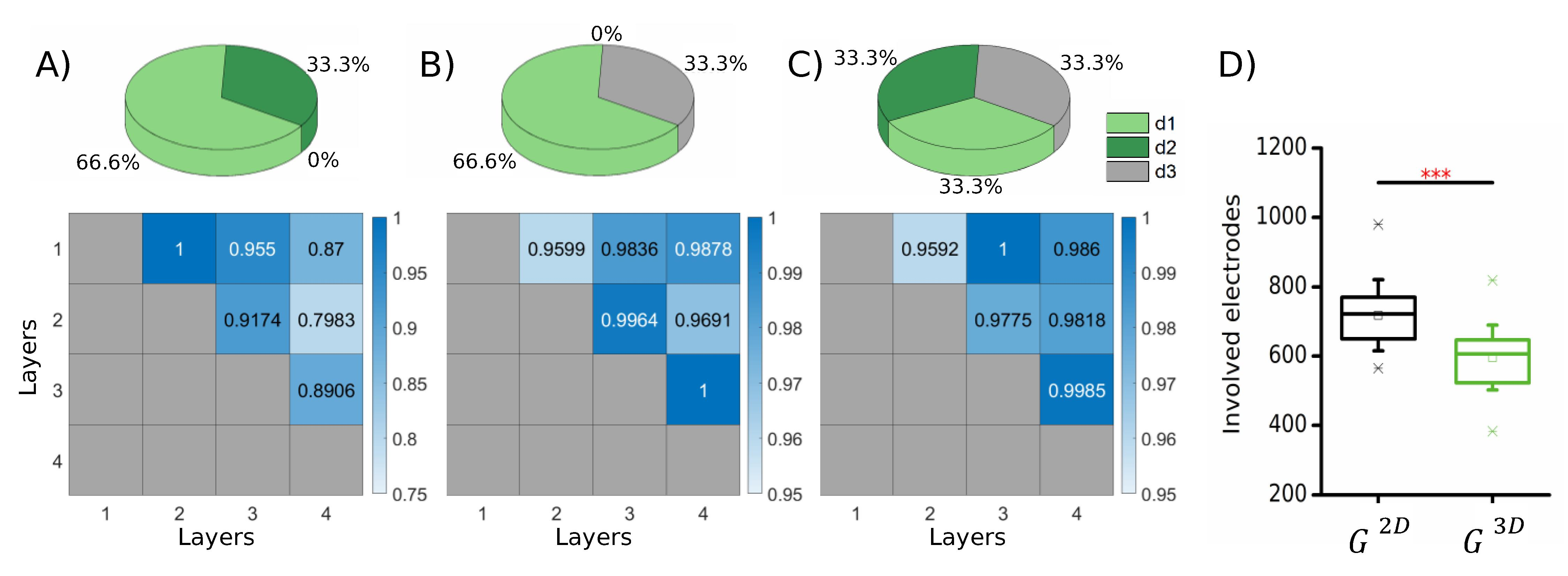

Supplement: S2 Fig — (A) Number of involved electrodes in the network burst events in 2D (black) and 3D (green) networks (*** refers to p<0.001, Kruskal-Wallis non-parametric test). (B-D) Color-coded normalized similarity maps, evaluated with the Victor Purpura distance, within 3 exemplary of 4-layer 3D networks. The normalization was done on the maximum similarity. From the maps, the three higher values of similarity were extracted and classified based on the step distance between two layers, (e.g., two consecutive layers are considered as 1-step distant; layer 1 and layer 4 are considered 3-step apart). Three different propagation modes emerged, each represented with an exemplary normalized similarity map and a relative pie chart, which indicates the percentage of times the higher similarity values occur between consecutive layers (d1), layers 2-step apart (d2), and layers 3-step apart (d3). Each identified propagation mode was exhibited by n = 3 simulated 4-layer networks (G3D). (TIF) [file pcbi.1010825.s002.tif]

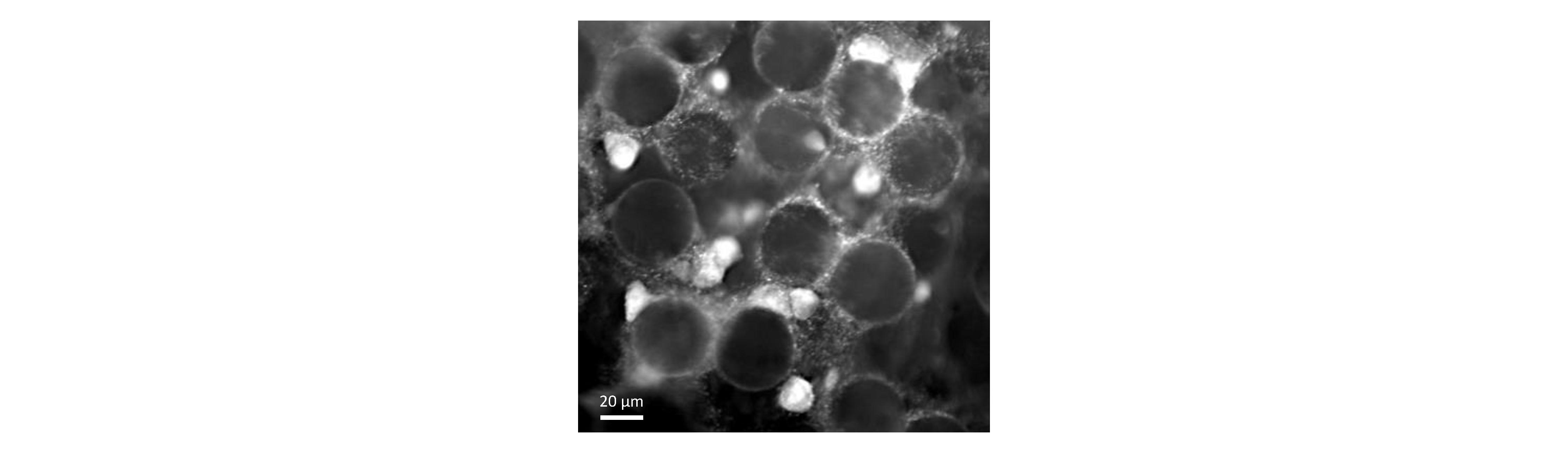

Supplement: S3 Fig — Mature 3D culture (18 DIV) on glass microbeads, stained with anti-NeuN antibody to label neuronal nuclear protein. (TIF) [file pcbi.1010825.s003.tif]

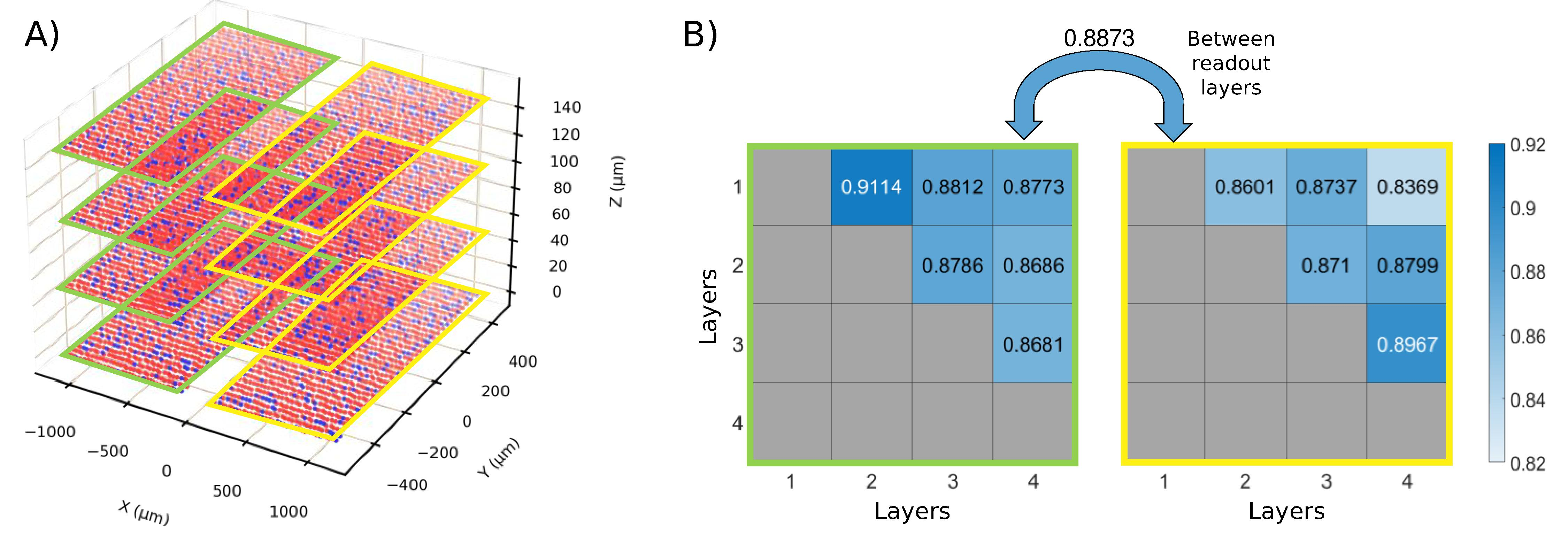

Supplement: S4 Fig — (A) representation of the disposition of the neurons in the network. Two different populations (green and yellow) were created as with the Gaussian connectivity (G2D and G3D). The two modules were connected on the readout layer level to mimic the experimental model devised in [13], where two different 3D populations were interconnected by microchannels. To imitate the physical constraints, the populations were 250 μm apart and the gaussian function that regulates the synaptic weight w and the probability of connection p was modified to introduce anisotropy. In particular, two different σ were implemented, a transversal one that is a multiple of the distance between two channels (50 μm) and a longitudinal one, that adds to that the distance between the two modules. (B) Color-coded similarity maps, evaluated with the Victor Purpura distance (S1 File), within the two modules (green on the left, yellow on the right). The arrow and value on the top indicate the similarity value between the readout layers of the two modules. (TIF) [file pcbi.1010825.s004.tif]

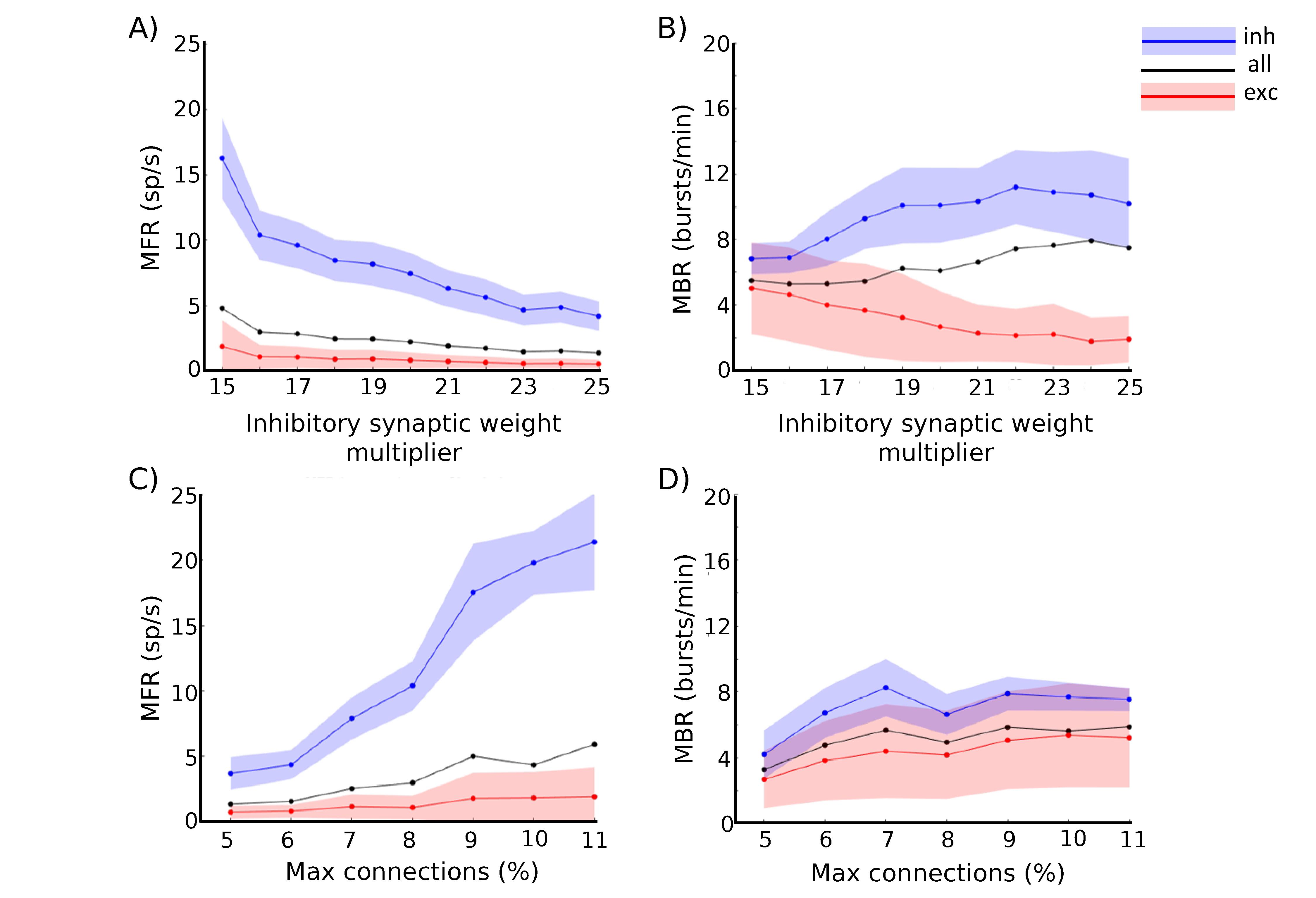

Supplement: S5 Fig — Effect of the mean synaptic inhibitory weights on the (A) mean firing rate (MFR) and (B) mean bursting rate (MBR). The effect of the percentage of pruning synaptic connections was evaluated and its effect evaluated on the (C) MFR and (D) MBR. Red, blue and black colors identify the results relative to the excitatory, inhibitory, and the total neurons, respectively. (TIF) [file pcbi.1010825.s005.tif]

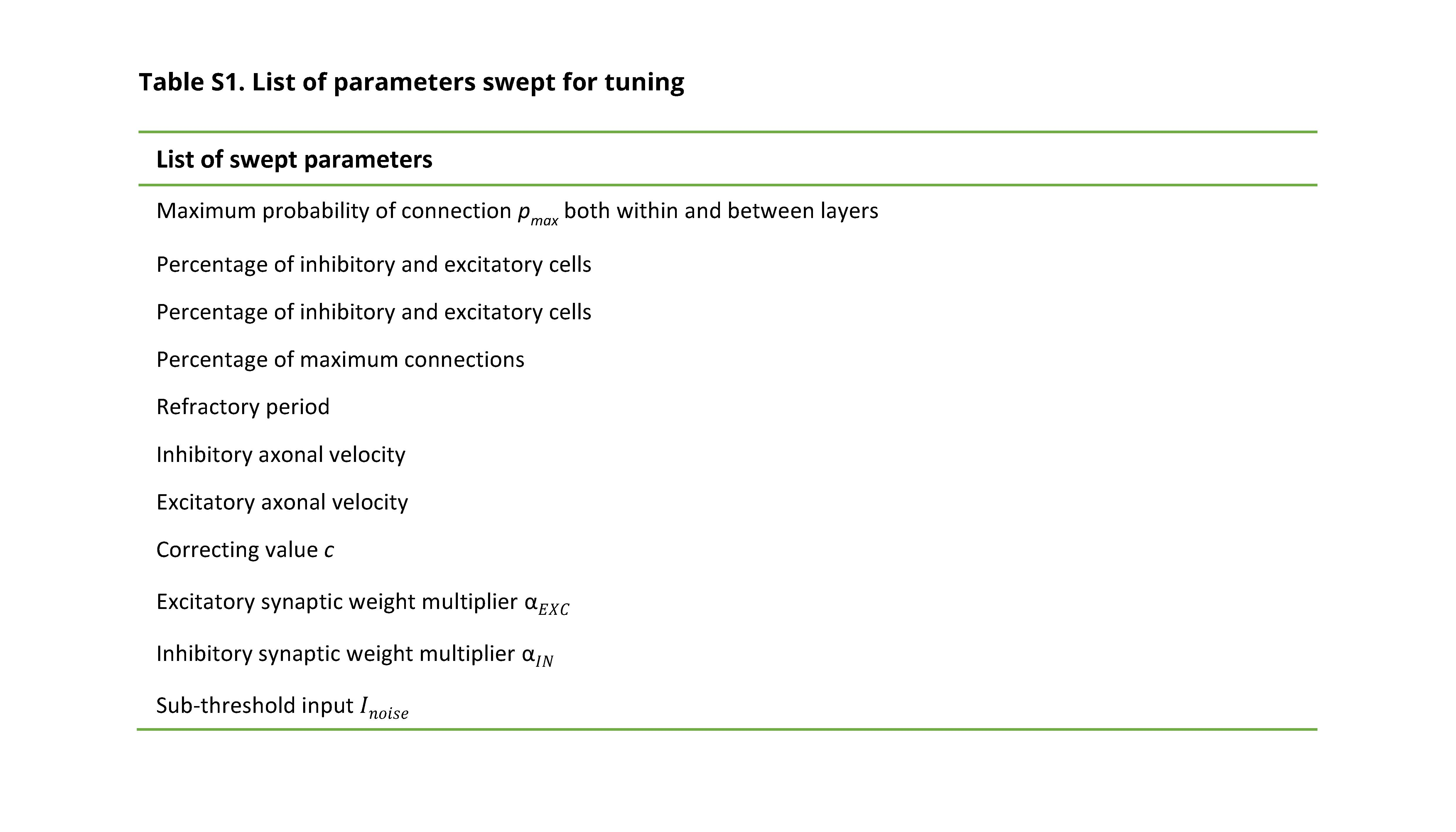

Supplement: S1 Table — (TIF) [file pcbi.1010825.s006.tif]
