# Supplementary material for: Modeling the three-dimensional connectivity of in vitro cortical ensembles coupled to Micro-Electrode Arrays
Source: PLoS Comput Biol. 2023 Feb 13;19(2):e1010825. doi: 10.1371/journal.pcbi.1010825 (PMC9956882; doi:10.1371/journal.pcbi.1010825)
Supplement: S1 File — Method for the extraction and evaluation of the similarity maps. (DOCX) [file pcbi.1010825.s007.docx]

**File S1. Evaluation of similarity method**

To compute the similarity between two populations, we considered the onset of the network burst events of each layer. After extracting the train of the onsets, we computed the normalized Victor Purpura [1] distance between the network burst trains of each layer. Briefly, the algorithm is based on finding the minimal cost for transforming the event train of one layer into the one of another layer by inserting, deleting (cost 1) and shifting (cost 0.5) the events. The distance was then normalized over the maximum possible cost to obtain values in the [0, 1] range, so that the values are comparable across simulations. The similarity value is computed as 1-cost. Similarity maps were then obtained in Matlab with as a color coded heatmap.

**Reference**

1. Victor JD, Purpura KP. Nature and precision of temporal coding in visual cortex: a metric-space analysis. https://doi.org/101152/jn19967621310. 1996;76: 1310–1326. doi:10.1152/JN.1996.76.2.1310
