# Supplementary material for: Modeling the three-dimensional connectivity of in vitro cortical ensembles coupled to Micro-Electrode Arrays
Source: PLoS Comput Biol. 2023 Feb 13;19(2):e1010825. doi: 10.1371/journal.pcbi.1010825 (PMC9956882; doi:10.1371/journal.pcbi.1010825)
Supplement: Attachment — Submitted filename: Answers-to-Reviewers_Final.pdf [file pcbi.1010825.s008.pdf]

**Reviewer #1**

This is an interesting article, well and written, and deserves publication in PLoS Computational Biology. However, I have some concerns that must be addressed before publication.

We thank the reviewer for the positive comment he gave on our work. Following his suggestions, we performed substantial revisions in order to make clearer and more readable the Introduction (especially in the context of functional/structural connectivity). The Results section has been completely revised too, with the aim to make it easier distinguishing the different simulated configurations. Finally, the Discussion has been expanded by pointing out the main limitations of the model in its current form.

1. The introduction is overall clear and introduces the problem well. However, authors could mention (either in the introduction or later in the discussion) the efforts made to understand the relation between structural and functional connectivity beyond the general reference 21. For instance, Ludl et al. (Front Comput Neurosci 2020) showed using numerical simulations that strong spatial constraints help dictating network dynamics and functional traits. Additionally, my impression (and I think consensus from the community) is that the relation between structure and function is not direct given the intrinsic nonlinear behavior of neuronal systems and the ubiquitous presence of noise. For instance, using calcium imaging, Yamamoto et al. (Sci Adv 2018) observed that networks designed structurally in a modular way could show very different dynamics and functionality depending on the coupling between the modules, suggesting that small changes in structural connectivity could render very different functional networks. Somehow these aspects have to be reflected in the manuscript. Otherwise, one gets the impression that structural connectivity can be predicted from just analyzing the dynamics.

   The reviewer got an important point in the genesis of our model and underlined a possible weak point of the approach we followed using functional connectivity to design the structural network at the basis of the model. We totally agree that the same structural connectivity can originate different functional networks since the emerging spontaneous activity can display different dynamical states (state-dependent connectivity). However, it is also worth mentioning that under a set of reasonable hypotheses, it is possible to predict structural networks from functional ones (e.g., sufficient long recordings to attenuate the effects of spontaneous fluctuations), and that the achieved result is strictly dependent on the computational strategy used to infer functional connectivity (e.g., correlation-based vs information theory-based algorithms). In the revised version of the Introduction, we toned down the statement "functional-predicts-structural", by contextualizing the working hypothesis and by describing possible limits in the followed approach (revised version of the Discussion). For the reviewer's convenience, we reported here below the added paragraphs.

   "*In addition, the use of optical instruments provides simultaneously information about the interplay between structural connectivity and the emerging activity. This last feature is of extreme interest when we deal with high density (2D and 3D) neuronal assemblies. In 2017, Marom and colleagues developed an optical-accessible in vitro model of 3D networks by injecting a genetically encoded calcium indicator [18] to probe the variations of the emerging electrophysiological activity under their natural development. Many the works in the literature dealing with in vitro cultures exploit functional connectivity hints to infer structural properties [19]. Indeed, functional connectivity is strongly influenced by how it is measured (correlation- vs information-theory based algorithms for example), and some caveats are required to translate functional features into structural ones. First, functional measures are subject to fluctuations if achieved from short periods of recordings. Theoretical studies demonstrated the requirement of at least 10 minutes of recordings to avoid unstable results [20]. In addition, the achieved functional properties of a network can be considered predictors of the structural ones, only at small-scale, i.e., considering small ensembles of neurons [21]. Finally, it is worth mentioning the state-dependency of the functional connectivity: in other words, the same structural connectivity can originate different functional networks which exhibit different topological features. Thus, taking into consideration the weak points of the approach, the correlation between structural and- functional connectivity can be considered is indeed a good indication toon infer*

*topological properties reciprocally, but taking into considerations the weak points of the approach. In this work, although knowing the weak points of the functional-structural relationships, we implemented precise structural connectivity rules in the computational model, driven by the inferred functional networks."*

*"From a connectivity point of view, the different structural configurations we implemented in the model are inspired by their relative functional counterparts. Indeed, this working hypothesis is based on the in vivo experimental findings that suggest the possibility to infer structural connections from the functional ones [21,22] at the level of small assemblies of neurons. We are aware that this approach introduces some limits in the validity of our model. It is also worth mentioning that functional topological features are strongly influenced by the detection algorithm, and the outcome is state-dependent. This last source of variability was clearly proved in in vivo sensory-motor tasks, where it was observed that the functional connectivity changes from resting state to task-induced conditions [42]. In in vitro assemblies, multiple modes of functional connectivity are much more limited because of the stereotyped patterns of activity that typically emerge from synaptic interactions [20] and the lack of external stimuli. A match between experimental and in silico structural connectivity could be achieved by modifying the experimental conditions of plating: a reduction of the cell density makes the identification of the network organization easier using microscopy techniques, and consequently could help the translation of the morphological observations to simulated connections."*

2. In Methods, the authors explain that they design 2D networks that are vertically coupled to shape the final 3D system. If I understand correctly, structural connectivity within each 2D layer is imposed to be scale free based on Ref. [18]. I found this worrisome, since Ref. [18] provides functional data for connectivity distributions, not structural. So somehow the authors are assuming that functional connectivity is a good representation of the structural one, which I think is not correct. At least it should be explained that a strong assumption is made. It is also difficult for me to understand why identical neurons that homogeneously cover a 2D area shape a scale free structural network without any kind of connectivity guidance or constraints.
We apologized to the reviewer for the lack of clarity in the Method Section. Scale-free connectivity is not the only one configuration we implemented, and we tested with the model. The reference connectivity rule we implemented in the 2D layers is based on a Gaussian distribution which rules the probability to establish connections with the distance. In the revised version of the manuscript, in the "2D Connectivity models" section, we rephrased the paragraph which describes the implemented scheme:

*"Different connectivity rules (inspired by the experimental findings on functional connectivity) were applied and studied within the fundamental 2D unit. In any of the following cases, autapses were avoided as they are unlikely to form in dissociated neuronal cultures [49]. Only excitatory to excitatory, excitatory to inhibitory, and inhibitory to excitatory connections were implemented in the present work driven by experimental findings. At the basis of all the connectivity implementations, a probabilistic connectivity rule was created as in the following. The probability to establish a connection between two neurons was modulated according to a Gaussian decay over distance:…"*

However, we cannot deny the choice to design alternative 2D connectivities to model the topological properties of the layers. Indeed, the choice of a scale-free connectivity is inspired by functional findings (not only the old ref. 18). As we wrote in the previous answer, we followed the approach "functional suggests structural". However, with respect to the original version of the manuscript, we better motivate and contextualize such an approach providing also the possible drawbacks.

3. Related to this, I note for instance that Orlandi et al (Nat. Phys 2013) showed numerically that a Gaussian-like structural connectivity reflects well the dynamic behavior of 2D experimental cultures. In their simulations they observed that scale free behavior emerged in the dynamics of the network

due to the interplay of connectivity, metric correlations and noise. Thus, I think that in the present article, the authors should test what happens when they just assume that structural connectivity in each 2D layer is Gaussian or just dictated by the distance-dependent connectivity rule, without imposing a scale free connectivity. My feeling is that the results will not change much, strengthening the importance of the interlayer connectivity.

We agree with the reviewer's observation. Indeed, we have taken into consideration that a Gaussian-like connectivity could give rise to different dynamical states and to the critical functional networks that are observed *in vitro*. In fact, most of the presented results of the 2D and the 3D networks are obtained with networks where the connections are created following a Gaussian probability over distance in the 3D space. In this condition, only a pruning over the maximum number of connections was implemented (keeping the Gaussian probability distribution) as a condition to modulate the spiking activity to avoid divergences. However, we wondered whether the observed scale-free functional connectivity in in vitro cultures was proxy to a scale-free topological organization. This question was investigated in Results' section "*Effect of the different connectivity rules at the single layer level*", where we compared the effects produced by this type of connectivity with the purely probabilistic one (Rule 1). The results indicate that the introduction of a scale-free connectivity in one layer (the bottom one) or in all the layers reduces the impact of the third dimension.

For the sake of clarity, the manuscript was greatly revised to highlight the simulation conditions for each paragraph. For the same reason, a new summarizing figure was added in order to make clearer kind of connectivity rules (2D and 3D) in each condition, as reported below:

| Description | | # sim |
|---|---|---|
| $G^{2D}$ | Gaussian connectivity rule in a single layer | 20 |
| $G^{3D}$ | Gaussian connectivity rule within every layer of the 3D population. | 25 |
| $G^{3D}_{rnd}$ | Gaussian connectivity rule within every layer of the 3D population. The neurons that project to the other layers (source neurons) are a random subset of the total neurons of each layer (Figure 6F) | 4 |
| $G^{3D}_{C}$ | Gaussian connectivity rule within every layer of the 3D population. The source neurons are a located in a central cluster of each layer (Figure 6G) | 4 |
| $G^{3D}_{5C}$ | Gaussian connectivity rule within every layer of the 3D population. The source neurons are a located in five clusters (Figure 6H) | 4 |
| $SF^{2D}$ | Scale Free connectivity rule in a single layer | 28 |
| $SF^{3D}_{all}$ | Scale Free connectivity rule within every layer of the 3D population. Each neuron could project afferences to the other layers. | 22 |
| $SF^{3D}_{L0}$ | Scale Free connectivity rule within only the readout (bottom, L0) layer of the 3D population. The connections within the other layers follow the Gaussian connectivity rule. Each neuron could project afferences to the other layers. | 12 |

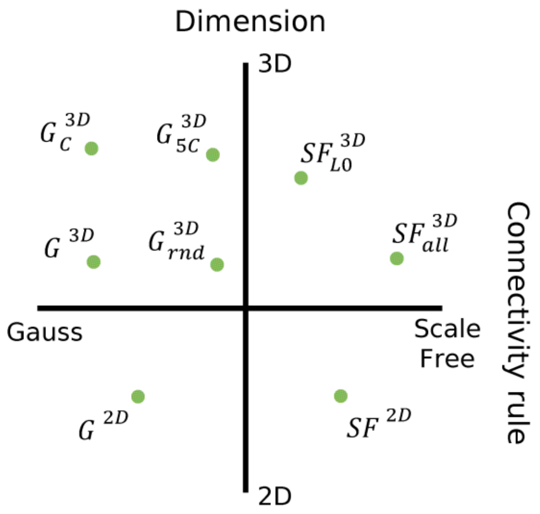


**Figure 7. Representation of the dataset.** All the configurations analyzed in the paper are reported here with their abbreviation and a brief description. For all the 3D, the connectivity between the layers follows a Gaussian distribution scaled over distance as reported in Sect. 3D connectivity.

4. Most of the panels in Fig. 1 are not necessary. There many works in the literature describing how network bursting increases as the inhibitory-to-excitatory ratio is reduced. What I missed in Fig. 1 are raster plots of the full 3D system comparing the interlayer connectivity schemes. I would expect to see that the different layers do not necessarily activate synchronously, which I think is an interesting message. The authors could then mark which of the layers in the 3D network raster plot correspond to the "MEA readout" layer that they wish to compare with experiments.

In the revised version of the manuscript, we took into account the reviewer's suggestion by drawing a "3D raster plot" in order to give the readers a compact overview of the 3D network activity. Thus, Figure 1 has been completely reorganized in order to display the activity over the different layers and, by means of the zoom of the network bursts (and the correspondent firing profiles), we extracted simple sketches of propagation schemes among the layers. For the reviewer's convenience the new Figure 1 (and the correspondent caption) was reported below.
The old Figure 1 was moved as Supplementary Information (now Fig. S1).

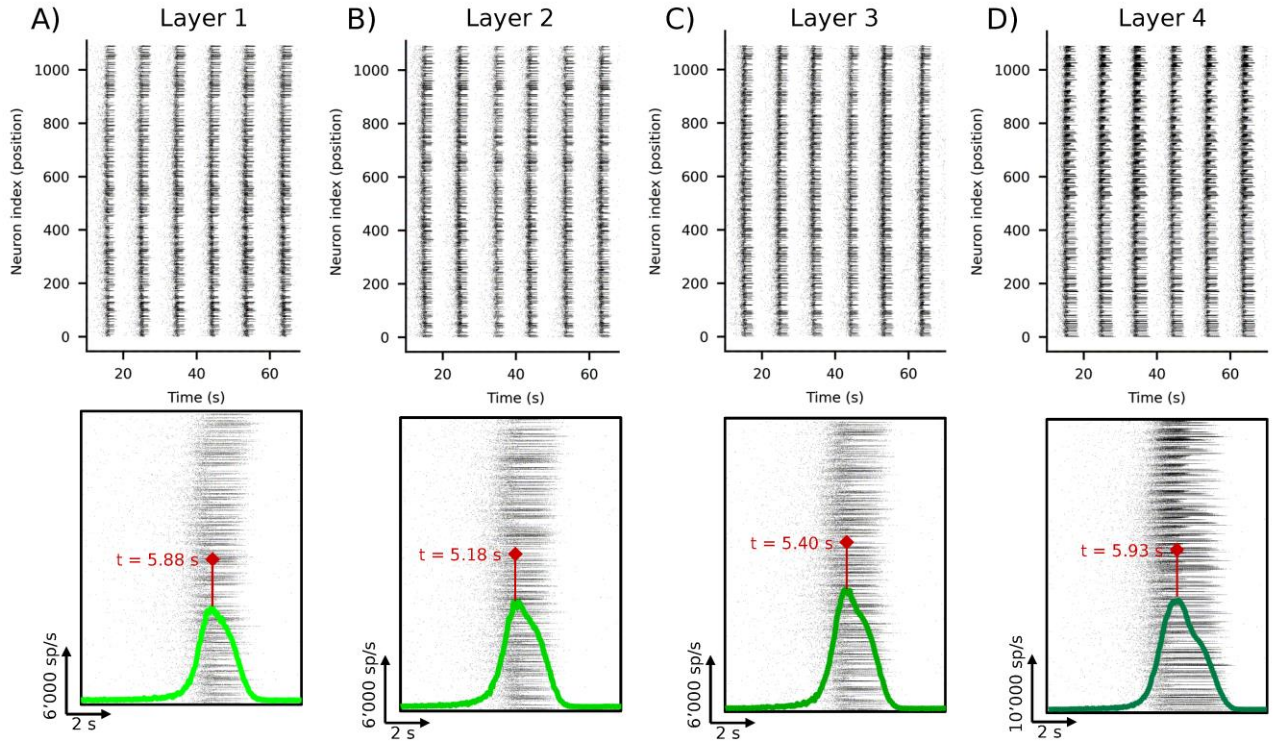


**Figure 1. Raster plots and relative IFR traces of the layers of a single population with Gaussian connectivity ($G^{3D}$) that guarantees *in vitro*-like patterns of electrophysiological activity where bursting and spiking activity coexist.** (***First row***) 60-s simulated spontaneous electrophysiological activity of the layers of a representative 4-layer 3D network (each panel **A-D** refers to a single layer of the population, with **A** being the bottom/readout layer)**.** This configuration allows to reproduce values of firing and bursting rates comparable with the experimental recordings of mature cortical cultures as well as the presence of population events (network bursts) involving most of the neurons of the network. (***Second row***) Close-up of a single population event with super-imposed IFR traces (***green***) and temporal stamps of the relative maxima (***red***).

5. I got confused in Fig. 4. The authors seem to suddenly show experimental data (since they mention in Methods that they have experimental data), but the discussion of the figure seems to be about simulations. I got lost. Can the authors put both experimental and numerical data in that panel? Additionally, in line 234-235, the sentence "As a tight correspondence between functional and structural connectivity was observed…" is very strong sentence if it refers to experimental data. Where is the proof that structural connectivity and functional one coincide?
We apologize for the confusion caused by the presence of separate experimental and numerical data. To better understand the manuscript, we decided to remove the uninformative Figure 4. The "pure experimental panel (old Figure 4C)" has been moved in the "Supplementary Information" section (Figure S4).
We also agree with the reviewer about the reported sentence that it is too strong. Indeed, in the present work, we have no claim to demonstrate the strong correspondence between structural and

functional connectivity. Based on the work of Bullmore and colleagues, the intent was to understand the functioning of three-dimensional networks starting from the experimental information (based on functional analyses) in our possession.

6. The discussion is nice, but the authors could extend it a little bit by commenting the limitations and extensions of their work. For instance, they could discuss on the possibility to break the synchronous bursting behavior by adding modularity in the 2D layers and by altering the range of interlayer connections, so some neurons can connect beyond their immediate neighborhood, somehow approaching the cortical columns observed in the brain. I have the feeling that the 3D system they obtain can be viewed as a 2D 'readout' network with effective bombardment of activity from the other 2D networks, which disrupts its collective behavior.
   We agree with the reviewer observation. Indeed, adding modularity as feature would further increase the reliability of the model. In this perspective, we decided to run a simulation of a network with two different 3D modules that can communicate at the bottom layer level. We evaluated the similarity of the network burst initiation events within the two modules and between the bottom layers. Indeed, we found out from this preliminary analysis that the communication between two modules produces a different effect on them. Focusing on the bottom layer, one of the modules shows higher inner similarity values than the one with the other module, suggesting that the signal propagates better to the higher levels. The readout layer of the second module however results to be more similar to the first module than to the other layers of its own module, suggesting that the propagation of the signal is greatly disrupted by the addition of modularity. Following the reviewer suggestion, we expanded the discussion and added these observations, as reported below:

   *"In perspective, the proposed in silico model could also be exploited to investigate other open questions related to 3D ensembles and can be customized for peculiar experimental setup. To prove such potentiality, we performed preliminary simulations of an experimental condition where (at least) two 3D neuronal ensembles are interconnected by means of bundles of links originating from the bottom layers (Fig. S4 A). In this way, in addition to the 3D connectivity, also the modularity topological feature was considered [45]. This configuration (also reproduced in in vitro experimental models) better mimics the large-scale network organization of mammalian brain and allows to reproduce a wider repertoire of dynamics [46]. Preliminary simulations show that from the similarity maps (Figure S4 B), we can observe that whilst one of the modules continues showing the highest similarity values within the module, the network burst initiation events of second module present lower inner similarity values than the ones obtained with the first module suggesting that the propagation of the signal is greatly disrupted by the addition of modularity."*

Other minor aspects:

1. Details are necessary in the description of the box plots of the figures. How many data points are in the distributions? Is the data coming from a single numerical repetition or several ones? Are results consistent among repetitions?
   We agree with the reviewer. To simplify the reading of the text, we added the "*Statistical analysis*" section, where we reported the information related to the graphs and the type of statistics carried out on the data. For the reviewer's convenience, here below we reported the content of this new section:

   *"Statistical analysis was performed using Origin (Origin Lab Northampton, Ma) with the non-parametric Kruskal–Wallis test, as data do not follow a normal distribution (evaluated by the Kolmogorov-Smirnov normality test). Significance levels were set at $p < 0.05$. The box plots representation indicates the 25–75 percentile (box), the standard deviation (whiskers), the mean (square), and the median (line) values. For the last section, to make the data comparable, different*

*subsets of the 3D reference configurations with dimensions comparable to the other data were tested, each resulting statistically different."*

In addition, in the "*Dataset*" section we added a new figure and a new table to highlight the number of simulations for each configuration, reported above.

2. In the caption of Fig. 7, it should be clarified that the distribution of panel E refers to a single 2D layer, not the entire 3D construction.
   The scale-free distribution has been implemented in different configurations, to better express the concept we changed the caption as follows:

   "*(**E**) Degree distribution of the scale-free network showing a power-law with characteristics exponent equal to -0.61, in accordance with [18]. The distribution was implemented either only in the readout layer, in all the layers or in none of them. The connectivity among the layers was modeled according to different possible configurations depicted in the panels*"

3. The caption of Fig. 2 should clarify that the 3D data is actually the 2D readout layer, not the whole 3D population.
   We agree with the reviewer. We modified the caption as follows:

   "***Electrophysiological features of 2D (black) and readout layer of 3D (green) networks.** (**A**) Mean firing rate, (**B**) mean bursting rate, (**C**) burst duration, (**D**) network burst duration. (**E**) Average Spike Times Histogram (STH) of a 2D and a 3D exemplary configuration. (**F**) Temporal time constants of the (**left**) rise and (**right**) decay phases of the network bursts. (* refers to 0.01<p<0.05, ** 0.001<p<0.01, *** p<0.001, Kruskal-Wallis non-parametric test)."*

4. Line 416: with characteristics exponent -> with characteristic exponent.
   We modified it.

5. It should be clearer in Fig. 4 that the shown data is 'in vitro'. Maybe in the title, replace 'cortical assemblies' by 'cortical assemblies in vitro'.
   In the revised version of the manuscript, we removed Figure 4.

**Reviewer #2**

The study of Callegari and colleagues is a 3D neuronal network model trying to simulate and reproduce the experimental conditions/data from Pautot and Frega papers. This is a very interesting topic, since 3D in-vitro neuronal networks are key experimental models overcoming previous 2D models (classical dissociated neuronal cultures) to study brain functioning and diseases. In-vitro 3D networks show broader repertoire of dynamics compared to 2D networks possibly better reproducing brain functioning mechanisms. 3D networks from dissociated neurons or brain organoids are the new frontiers of in-vitro brain models. 3D networks models reproducing 3D cultures are still lacking, therefore Callegari et all focus and explore a high interest topic both for computational and experimental neuroscientists. The main result is that the introduction of three-dimensionality induces a decrease in both firing and bursting rates with respect to 2D models. In addition, the authors found that at least four layers are necessary to induce a change in the dynamical state of the network.
We thank the reviewer for his/her positive comment and for having captured the goal and the aims of the developed model. We made a systematic revision of the entire manuscript trying to fix both the textual as well as the computational concerns.

My first major comment is that the there is no mention in the model and in the experimental data on the rich dynamical regimes observed in the 3D case. I mean 3D are better than 2D networks because dynamics are different and not only in terms of single neuron and network firing and burst rates, but also in term of terms of neural synchronizations. The 3D network display dynamics which go beyond the stereotyped 2D networks activity characterized by network bursts which recruit the overall circuit. This is a key aspect which should be also addressed in the work.
The reviewer is right, the activity of 3D networks shows a richer repertoire of dynamical states then 2D. However, the general propagation between the layers goes a little beyond the scope of the current work. In fact, our work focuses on reproducing the experimental data obtained with planar MEAs, which give no indication on the propagation between the layers. For this reason, we did not particularly focus on the synchronization of the layers. However, we agree with the reviewer on the importance of this feature, and we included in the revised version of the manuscript some considerations on the following parameters: the delays in the peak activation of the network bursts, obtained from the IFR traces (Figure 1, second row), the similarity values between the activation times of the network bursts of different layers (Supplementary information, Figure S2), and the involvement of the nodes (neurons) in the population events (Supplementary information, Figure S2). Also, we added a paragraph discussing the propagation in the results, as reported below, and added a Supplementary Information File describing the methods for the extraction of the similarity maps.

*"The result of the chosen combined parameters of these networks (Table 1) gave rise to a sustained spiking and bursting activity also in the 3D configuration, with network bursts that involved almost the entire network, as shown by the 60-s raster plots of a 4-layer network in Figure 1A-D (and the respective close-ups of a single event in the lower row). Considering the population events, we considered their propagation among the layers. Qualitatively, the IFR traces reported in Figure 1 (lower row) highlight the time stamp of the peak associated with one of the network bursts. From these values, we inferred the delay in the propagation of the event between the layers. In particular, this representative event started from the second layer, propagated to the third and the first ones and only then arrived to the fourth layer, that is the most active. In general, we observed a high variability in the generation and propagation of the network events (as demonstrated by the similarity maps of Fig S2 B-D, obtained with the Victor-Purpora statistics [27] described in the Supplementary Information File S1), possibly brought by the intricate connections established in the 3D space. Moreover, we observed that the number of involved neurons during these events significantly decreases if compared to 2D networks (Fig S2 A). This fact suggests that the stereotyped activity of the network is on some level severed by the introduction of the third dimension, as a lower percentage of cells is involved in the network event.*
*However, considering the higher level of complexity in the evaluation of the whole network and the lack of an in vitro experimental counterpart (data from planar MEAs), the focus of our analysis was on the examination*

*of the metrics obtained only from the bottom (or readout) layer of 3D networks and from 2D ones to try inferring some differences based solely on this reduced observation point of view."*

My other main major comment is that in the actual state, the paper is extremely difficult to be read and understood, and it requires a very challenging ping-pong between the methods, the main studies to which the work refer to (Frega et all, Pautot et all), and the data shown in fig 4 (are the results from another paper or generated and freshly within this research?).
We thank the reviewer for the observation. Following his suggestion, we greatly revised the manuscript in order to ease its reading. In particular, we made the following changes:

- We introduced a paragraph at the beginning of each result section stating and summarizing the conditions/parameters of the simulations for that section
- We introduced Figure 7, that summarizes the used dataset (reported below).
- We added a brief description of the literature methods where they are cited

Regarding the experimental results of figure 4 and 6, we extracted the data with experimental sessions in our lab. A focused paper on the experimental results was published in Brofiga et al. (2020), other results were extracted anew (the data is included in the dataset).

Also, the introduction and the paragraph on the scale-free connectivity is very hard to be understood. What are the four conditions considered? Same story when the scale-free 2D networks results are compared to SFALL and SF0. The high mix of data and models with lack of proper explanation undermine the richness of the available experimental data (2D and 3D).
We agree with the reviewer's observation. In this work, we tested many connectivity configurations trying to resemble the *in vitro* one(s). Indeed, we agree with the reviewer's observation that the different combinations of implemented connectivity are not "easy" to follow in the manuscript reading. Thus, to facilitate the flow of the manuscript, we added some summary sentences at the beginning of each paragraph to explain the conditions analyzed. In addition, we also included a summary table of all the conditions used in the "Dataset" section. Also, the acronyms used have been revised in order to make them easier.

For the sake of the reviewer's simplicity, we report the new figure below.

| **Description** | | **# sim** |
|---|---|---|
| $G^{2D}$ | Gaussian connectivity rule in a single layer | 20 |
| $G^{3D}$ | Gaussian connectivity rule within every layer of the 3D population. | 25 |
| $G^{3D}_{rnd}$ | Gaussian connectivity rule within every layer of the 3D population. The neurons that project to the other layers (source neurons) are a random subset of the total neurons of each layer (Figure 6F) | 4 |
| $G^{3D}_{C}$ | Gaussian connectivity rule within every layer of the 3D population. The source neurons are a located in a central cluster of each layer (Figure 6G) | 4 |
| $G^{3D}_{5C}$ | Gaussian connectivity rule within every layer of the 3D population. The source neurons are a located in five clusters (Figure 6H) | 4 |
| $SF^{2D}$ | Scale Free connectivity rule in a single layer | 28 |
| $SF^{3D}_{all}$ | Scale Free connectivity rule within every layer of the 3D population. Each neuron could project afferences to the other layers. | 22 |
| $SF^{3D}_{L0}$ | Scale Free connectivity rule within only the readout (bottom, L0) layer of the 3D population. The connections within the other layers follow the Gaussian connectivity rule. Each neuron could project afferences to the other layers. | 12 |

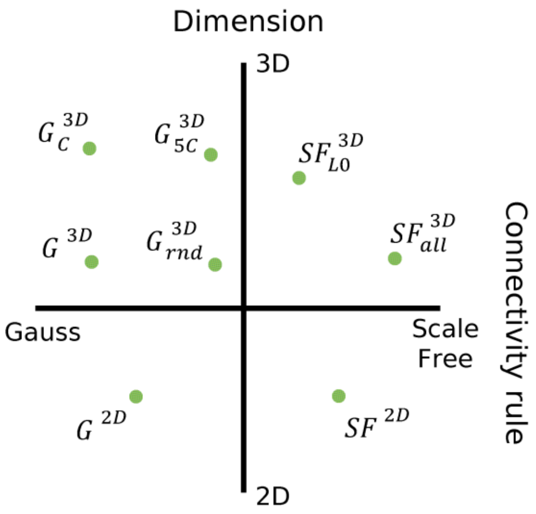


**Figure 7. Representation of the dataset.** All the configurations analyzed in the paper are reported here with

their abbreviation and a brief description. For all the 3D, the connectivity between the layers follows a Gaussian distribution scaled over distance as reported in Sect. 3D connectivity.

So, a major reshape of the MS is definitely needed. The model and its configurations cannot be only presented in the methods section. More details are needed within the MS especially in the opening part of the results, where all that is written now is confusing rather than helping to understand what is later presented.
More detailed comments follow, but a drastic reshape of the MS is needed to explain exactly how the model is built and which configurations are tested and to which experimental data you refer to.
We thank the reviewer for the observation. Following his/her suggestion, we greatly revised the manuscript in order to ease the reading and understanding of the manuscript as listed above.

ABSTRACT

1. "In addition, we found that at least four layers are necessary to induce a change in the dynamical state of the network and that a scale-free connectivity within a layer tends to reduce the effects of the 3D organization." this sentence is disturbing in the abstract, make it not clear in a way. Also, this refers only to the bottom layer as scale free? so it is not a general within layer organization.
   Following the reviewer's suggestion, we modified the abstract to address the doubts and to make it clearer. Indeed, we wanted to convey the observation that in general the scale-free connectivity tends to reduce the effect produced by a 3D structure, either if it is implemented in only one layer (in particular in the bottom one) or in all the layers. For the reviewer's convenience, we report the modified text:

   *"In addition, we found that there is a minimum number of layers necessary to obtain a change in the dynamics of the network. However, the effects produced by a 3D organization of the cells is somewhat mitigated if a scale-free connectivity is implemented in either one or all the layers of the network"*

INTRO

1. Line 68 to 74
   Self-citations are fine when also a broad view is presented. Most likely the experimental model you mentioned, and your following work (Frega et al) were the first works on 3D cultures and recording of their dynamics. However, I find this self-citation too strong in terms of relevance in the beginning of the intro. I suggest to smooth it or narrow to this point later.
   Indeed, you have many more ways to create 3D networks from dissociated neurons, so you should more broadly introduce them also in relation to their dynamic regimes. do 3D networks from dissociated neurons exhibit the same dynamical regime you try to reproduce? address this in intro to make your study of more broad interest. Otherwise, this narrows too much the interest to the Frega et all experimental model, limiting the interest of your work to only that specific 3D model. i think the study here (https://www.ncbi.nlm.nih.gov/pmc/articles/PMC5220075/) is an example of how 3D network regimes are different from 2D in line with the Frega paper results. Also the mentioned study uses a non constrained spatial configuration as the one imposed by the beads and also the mentioned study is based on optical methodolgy which offers single cell resolution, so makes the overall view non limited to electrical recordings. Please broaden your intro to make it of more general interest.
   We agree with the reviewer's observation. In the revised version of the manuscript, we re-arranged the experimental 3D scaffolding methods including other strategies to realize 3D assemblies to finally converge with the Pautot's method. Indeed, we emphasized pros and cons of the methods in terms of feasibility, reproducibility, and likelihood with the *in vivo* 3D structures. Here below, for the reviewer's convenience, we reported the new paragraphs:

   *"3D electrospun polymers or hydrogels are possible solutions to reach such a goal. Hydrogels ensure low cytotoxicity and favor exchange of gases and nutrients between cells and the surrounding environment. Collagen is another natural soft material that was used as a scaffold to develop 3D*

*neuronal assemblies. In 2015, a model of 3D brain-like tissue was realized by means of silk-collagen proteins [11], where neurons plated in a donut-shaped porous silk sponge were able to develop robust projections within the central region full of collagen, generating dense 3D neuronal networks. The principal drawback of many hydrogel-based materials is their relatively fast mechanical degradation. To overcome this weakness, in 2015, Bosi and colleagues developed a synthetic biocompatible PDMS scaffold that can be tuned in its micro and nanomechanical properties [12]. Unfortunately, this type of structure hinders the recording of electrophysiological activity. The use of planar MEAs would be, in fact, useless considering that most of the electrodes would be covered by the PDMS*"

2. Line 84-92

maybe discuss other electrophysiological methods to record 3D cultures. as I suggested before calcium imaging and optical measurement should be in principle able to record at different depth, with single cell resolution, overcoming intrinsic planar electrode array limitations (lack of information in terms of recording of cell location and type). In general the work you present is too narrowed to the papers of Frega, Bosi and Pautot. These are just starting points and you want to offer broader message indeed.

The choice to introduce and discuss only the experimental works of Pautot, Frega, and Bosi is ruled by the fact that the computational model is inspired (e.g., layer organization) by these experimental solutions. We agree with the reviewer's observation that the literature offers a wider choice of methods to realize 3D networks as well as to record their electrophysiological activity. We carefully considered his/her suggestion, and in the revised version of the manuscript, we decided to:

i) add in the Introduction section a short survey of other experimental set-up for recording the electrophysiological activity;

ii) add other "scaffolding" methods (not too far to the Pautot's one) to realize dense 3D assemblies (cf., previous answer);

iii) add in the Discussion a new section describing how the developed computational model can be adapted and generalized for other 3D techniques (the ones presented in the Introduction). For the reviewer's convenience, we reported here below the new paragraphs:

- In the introduction:

*"However, MEAs are not the only technological devices to record the electrophysiological activity of 3D neuronal networks: optical methods are a valid alternative solution to overcome such limitations: in contrast to MEA, optical-imaging-base d techniques using dyes or genetically encoded reporter constructs guarantee a higher spatial resolution and recordings at single cell resolution. Among these, calcium-imaging also allows to simplify the analysis of emerging functional activity, avoiding the use of spike sorting algorithms to discern the activity of dense clusters of 3D neurons recorded by planar or 3D electrodes. In addition, the use of optical instruments provides simultaneously information about the interplay between structural connectivity and the emerging activity. This last feature is of extreme interest when we deal with high density (2D and 3D) neuronal assemblies. In 2017, Marom and colleagues developed an optical-accessible in vitro model of 3D networks by injecting a genetically encoded calcium indicator [18] to probe the variations of the emerging electrophysiological activity under their natural development."*

- In the discussion:

*"In this work, we developed a computational model of a 3D neuronal network driven by a peculiar experimental configuration achieved by stacking (ordered) layers of neurons [43]. As we wrote in the Introduction, this is not the only method used to build in vitro 3D networks, and more and more studies are exploiting the use of soft and amorphous materials as scaffold to allow the 3D neuritic growth [5]. Indeed, a change in the biological organization of the network requires variations in the in silico model too. However, the structure of the current model allows a relativly easy modification of parameters like the cell density and neuron position in the 3D space to accomplish the experimental requirements. On the other way round, the design of new connectivity patterns could be not trivial.*

*An anisotropic scheme of connectivity will be necessary to map the inhomogeneity of the space where neurons live [44].”*

3. Line 102-104
   explain better, not clear, what you mean average length? spatial? mean path length is topological, not spatial. so better in case you clarify which length you refer to and make it clear. clustering, is spatial clustering or topological clustering? Please make this more clear just to better interpret the model, since you spend some sentences over it and you make clear focus on it
   We apologize for the lack of clarity in the description of these metrics. Lines 102-104 of the irst version of the manuscript were related to the paper published by Bosi and co-workers that extracted such metrics in their topological meaning (whose definitions are explained in the paper “Bullmore, E. & Sporns, O. Complex brain networks: graph theoretical analysis of structural and functional systems. Nat Rev Neurosci. 10, 186–198 (2009)”) compared to the geometrical length of the simulated connections. In our manuscript, we did not include a detailed description of such metrics since they were not used to characterize our simulations. However, to make that sentence more comprehensible in the Introduction, we rephrased as follows:

   *“After matching the inter-burst interval values originated by the simulations of the 2D and 3D networks to the ones derived from calcium imaging techniques, the authors characterized the results of their simulations in terms of the topological feature [22] of the network. They found a simultaneous increase of the clustering coefficient and no significant variations in the mean path length of the 3D networks, although the average geometrical length of the links increased [12].”*

4. line 110-113
   not clear that you refer to your present work
   Following the reviewer’s suggestion, we modified the sentence as follows:

   *“To design the in silico model of 3D networks, in the present work we took inspirations from the 2D topological organization (i.e., the one relative to the layer directed coupled to the active area of the MEA), inferred with functional connectivity investigations in dissociated 2D networks [19,20,23]."*

5. line 117-119
   which in vitro models, 3d or 2d? the last part of the introduction summarizing the work is not clear at all. I mean obviously after you read the paper it becomes better understandable, but as an intro which should facilitate to approach the overall work, surely this is not clear
   We thank the reviewer for the observation. We greatly revised the paragraph indicated by the reviewer as in the following to make it more suitable as introduction.

   *“Considering the experimental evidence of a scale-free functional connectivity observed in in vitro cultures, we explored the possibility of a structural implementation of this feature. We investigated the effect brought by the modification of the topological organization with a scale-free structural connectivity, finding that the SF topology limits the overall impact of the third dimension on the network. Eventually, the results were compared with those obtained from 3D in vitro cortical cultures, highlighting the relevance and the power of our in silico model”.*

RESULTS

1. At the beginning of the results, you have to better explain, as in the introduction, that you use as fundamental unit your model of 2D networks (single layer) and you next interconnect layers. Also the bottom layer is spatially unconstrained so it is the only layer that you also model it as a scale-free network (apart the case of SFall). All other layers are added next. Interconnectivity between layers is free or spatially constrained to neurons located in specific areas (two conditions are modelled). All this has to be clear at the beginning of the results, since this is the core of your model, so most of this part can be moved from methods to results section indeed.

We apologize for the lack of clarity. As suggested, the manuscript was greatly revised in the Results section as in the Introduction to better explain the conditions of the models and to underline which variables were examined. We report below the revised beginning of the results. Moreover, before each result is shown a summary of the conditions under which it is obtained.

*"In this section, results from the variation of the different constitutive parameters of the 3D neuronal model are presented and compared in order to unravel the contribution of each in the genesis of the spontaneous electrophysiological activity. Generally, the model consists of a spiking network of Leaky-Integrate and Fire (LIF) excitatory and inhibitory neurons arranged in layers with a density comparable to 2D in vitro cultures. These layers constitute the core element of the model, and they were piled up to create a 3D structure. To create a functional network, different connectivity patterns were implemented, guided by experimental evidence. The reference implemented connectivity both within and between the layers follows a gaussian probability ($G^{2D}$, $G^{3D}$) over distance with a limitation over the maximum number of established connections (see Materials and Methods for further details). Firstly, we examined the intrinsic dynamics of the this reference generated model and tested the effect of the 3D configuration on the activity of the network by comparing it to 2D structures whose connectivity is ruled by a Gaussian connectivity ($G^{2D}$) as well. Then, we investigated the effect of the number of layers defining the 3D networks to assess which configuration starts producing a significant change in the electrophysiological activity moving from a 2D to a 3D configuration. In this way, we were able to determine the height (i.e., number of layers) at which the model could be functionally considered a 3D structure. Next, Then, the role influence of the implemented established structural connectivity within each layer was explored, to test whether the functional characteristics observed in MEA recordings underlie an effective connectivity also in the 3D configuration. In particular, the functional scale-free (SF) connectivity described inferred in in vitro studies was implemented replicated in a structural connectivity fashion either in only the bottom layer (that as of now is the only one that has been observed in vitro, ($SF^{3D}_{L0}$) or in all the layers of the model ($SF^{3D}_{all}$). A similar investigation was devised for analysis was carried out on the inter-layer connections with the rationale of inferring the in vitro connectivity from the activity patterns recorded only at the readout (bottom) layer. In this case, different motifs of the position of the source neurons were conceived, with inter-layer connections generating either in clusters ($G^{3D}_{C}$ and $G^{3D}_{5C}$) or from random areas ($G^{3D}_{rnd}$). Eventually, the data of the last different configurations were compared to in vitro data of 3D cultures recorded by means of MEAs. At the beginning of each section below, the conditions of the simulations are summarized for the sake of clarity. Unless otherwise stated, in all the following analyses, the presented data are relative to the activity of the bottom layer (readout layer), to emulate the experimental condition achieved by using of planar MEAs."*

2. <u>Line 125-126</u>
"Firstly, we examined the intrinsic dynamics of the generated 126 model and tested the effect of the 3D configuration on the activity of the network." What do you mean here? This is totally unclear
We apologize for the lack of clarity. Indeed, we realized that, since the Materials and Methods is placed at the end of the paper, it is necessary to anticipate and explain the examined conditions at the beginning of each Section. Consequently, a recap paragraph of the dataset was added before each result. Moreover, an overall summary of the examined conditions was added at the beginning of the results (as reported above).

3. <u>Line 130-134</u>
"Then, the role of the established connectivity within each layer was explored, to test whether the functional characteristics observed in MEA recordings underlie an effective connectivity also in the 3D configuration. A similar analysis was carried out on the inter-layer connections with the rationale of inferring the in vitro connectivity from the activity patterns recorded at the readout layer. Eventually, the data of the different configurations were compared to in vitro data." This is not understandable to a person that did not read the paper. This means that is useless in the present

way. So as I suggested the first part of the results has to explain the model and the steps you will go over in the results.

We thank the reviewer for the observation. The result section was greatly revised, as reported above.

4. Figure 1

The only clear raster plot of fig 1 is the panel E. The rest of the rasters are not really visible. Indeed panel D is a black rectangle. Qualitative figures are ok, but still a better visualization of the different regimes has to be achieved. If not there is no real point to show the rasters. When you use MFR and MBR in the results for the first time, there is no mention of their abbreviation and meaning.

We thank the reviewer for the important observation. Indeed, the qualitative raster plots are less meaningful than what we intended, especially in low resolution versions. Therefore, we decided to move Figure 1 to the Supplementary Information (Figure S1) and replace it with a new Figure reported below. The new Figure 1 shows raster plots and the instantaneous firing rate (IFR) traces of the 3D refence configuration. As for the use of the abbreviations, we addressed the oversight and added their meanings upon first appearance.

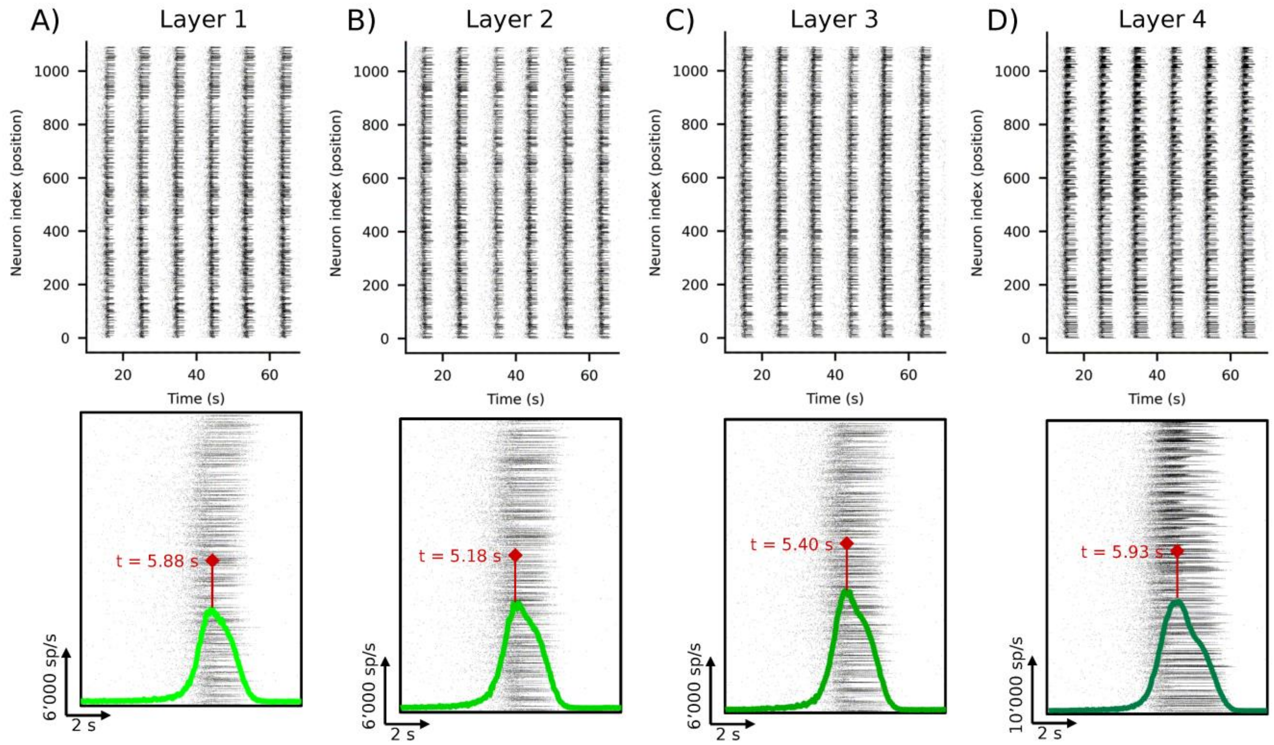


**Figure 1. Raster plots and relative IFR traces of the layers of a single population with Gaussian connectivity ($G^{3D}$) that guarantees *in vitro*-like patterns of electrophysiological activity where bursting and spiking activity coexist.** (***First row***) 60-s simulated spontaneous electrophysiological activity of the layers of a representative 4-layer 3D network (each panel **A-D** refers to a single layer of the population, with **A** being the bottom/readout layer)**.** This configuration allows to reproduce values of firing and bursting rates comparable with the experimental recordings of mature cortical cultures as well as the presence of population events (network bursts) involving most of the neurons of the network. (***Second row***) Close-up of a single population event with super-imposed IFR traces (***green***) and temporal stamps of the relative maxima (***red***).

5. Line 179

here you use RULE 1? say it clearly and not in between brackets in the previous sentence. also say what connectivity you establish between the layers, do you have similar rules to within layer? All this is defined in methods but you use also simply mention in the results. As I said part of the material you put in methods, it is indeed relevant to move it in the result, since it builds your model. Strictly

speaking only the LIF neurons and parametres used are for the methodological section. Topology within layer and inter-layer connectivity is your model and is needed a clear explanation in the result section.

The configuration of the networks employed in the section is the reference one, where the connections both within and between the layers are established following a Gaussian probability over distance. To clarify the conditions in the paragraph, it was greatly revised as in the following:

*"The 3D network of our model was built using the 2D fundamental unit as constitutive element. We tried to mimic the experimental construction of a 3D network with glass microbeads as scaffold. Briefly, as in Pautot's and Tesesco's works [7,9], the neuronal 3D cultures were created by plating the cells on the glass microbeads, then the cells and beads suspension was transferred to the MEAs, where they self-assemble in stacked layers (details in Sect In vitro model). In the computational model, this process was reproduced by piling up different 2D units (details and parameters can be found in the Materials and Methods). As dataset for the 3D configurations, we considered all the data obtained from simulations where a structural 3D network was created (2 up to 6 layers) with the reference connectivity rules (Figure 7, $G^{3D}$). That means that the connections both within and between the layers are established following a Gaussian probability over distance. The 3D data was compared against 2D networks with the same Gaussian connectivity rule within the layer (Figure 7, $G^{2D}$). "*

As for the suggestion to move a great part of the methods to the result section, we do not totally agree. Indeed, the manuscript had to be greatly revised to be clearer to the reader as was recommended, but we believe that fragmenting the explanation of the model and dividing it among the Results and the Materials and Methods would be equally confusing. Indeed, we opted to revise the Introduction and the Results in a way that we hope is clearer to readers that have not yet read the whole paper, but we kept the thorough explanation and the details of the models in the Materials and Methods section to have an organic overall explanation.

6. Figure 2
   is this only 1 layer (2D) vs how many layers? details are missing in results and legend to make it clear.
   The simulation conditions of the paragraph and the figure to which the reviewer is referring to were rephrased to be clearer for the reader. We report the revised version for the reviewer's convenience:

   *"As dataset for the 3D configurations, we considered all the data obtained from simulations where a structural 3D network was created (2 up to 6 layers) with the reference connectivity rules ( $G^{3D}$). That means that the connections both within and between the layers are established following a Gaussian probability over distance. The 3D data was compared against 2D networks with the same Gaussian connectivity rule within the layer ($G^{2D}$)"*

7. Line 185-188
   "No significant variations were observed in the shape of the network bursts, both in the rise (Fig 2F, left) and in the decay (Fig 2F, right) phases, suggesting that the addition of the third dimension itself is not a sufficient condition to shape the collective behavior of the neuronal network." I mean you just show that 3D networks behave collectively differently in terms of NBBD, so now you contradict what you just said
   We agree with the reviewer's observation, the sentence is ill-written as it is. We were referring to the parameters that characterize the shape of the network burst in the rise and decay phases. Indeed, the $\tau$ values, that rule the steepness of the slopes, do not change among the different configurations. What changes is the duration of the network bursts (NBD), which, if combined with the previous information, implies a difference in the length of the plateau of the Network Burst. Two corrections were made in the manuscript following the reviewer's objection: firstly, we modified Figure 2E, so that the fitting limits for the rise and the decay phases are clearer to the reader; secondly, we modified the cited sentence as follows:

*“No significant variations were observed in the rise (Fig 2F, left) and in the decay (Fig 2F, right) phases of the network burst, suggesting that the addition of the third dimension itself is not a sufficient condition to modify the recruitment and deactivation time of the collective behavior of the neuronal network, it affects just its duration”*

8. Line 188-191
   “These results are in line with the considerations reported in [12], where 189 it was proposed that the key parameter in the modulation of the network burst dynamics is the alteration 190 of the E/I balance in the 3D connectivity due to different interacting populations (cortical and hippocampal 191 in that study).” Move this to discussion, this is confusing especially when different source circuits are mentioned (hippocampus and cortex)
   Following the reviewer’s suggestion, we moved the sentence in the discussion as follows:

   *“Moreover, although the dynamics of the network is modified by the more complex 3D organization, the recruitment and deactivation time of the collective behavior of the neuronal network are not affected. These results are in line with the considerations reported in [13], where it was proposed that the key parameter in the modulation of the network burst dynamics is the alteration of the E/I balance in the 3D connectivity due to different interacting populations (cortical and hippocampal in that study).”*

9. Line 232-239
   this is not clear. if you rely on previous measurements, then you have to better summarize the result without the need to interrupt the reading of this paper and read the paper [21] to be able to advance. how inhibitory and excitatory connections were extracted? at least a mention is needed. Also you show here results from 2D networks, not3D, so be clear because if not it is confusing
   In the reported sentence, we refer both to previous measurements and to conclusions found in the literature. The inhibitory and excitatory connections were inferred from the cross-correlation as reported in the literature [34]. Briefly, the algorithm assumes that excitatory (inhibitory) interactions cause a spike rate increase (decrease). Consequently, the cross-correlogram shows a maximum (minimum) followed by low values modulated by the refractory period associated with excitatory (inhibitory) links.
   To avoid unnecessary confusion and considering the relatively uninformative Figure 4, we decided to remove it and to add only the panel relative to the 3D cultures that inspired the model in the supplementary information (Figure S3).

10. Line 236
    in the literature, from this review, the match between structure and function has been more and more shifted to the modular level. so structural-functional match on single link is not really observed and relevant, because structural-functional modules emerge with no real single link match. This has to be taken into account addressed in the work when mentioning this pioneering paper.
    We thank the reviewer for this fundamental observation regarding the approach we followed in the design of our computational model. The relationship between structural vs functional connectivity has been completely revised in the new version of the Introduction, trying to better explain in which conditions the results coming from functional analysis can be predictive of the structural ones. We know the aforementioned work of Bullmore and Sporns (2009) was conceived for the functional/structural connections of large-scale areas, and the detected connections are not single links, but bundles of connections. However, the results achieved in that paper inspired the analyses of the interplay between structural and functional connectivity in *in vitro* neuronal networks (Poli, et al., J. Neur. Eng., 2016; Maccione et al., J. Neurosci. Meth., 2012; Napoli, et al., BMC Neurosci., 2014), demonstrating the validity and scalability of the approach. However, we understand the observation of the reviewer since in the original version of the manuscript such an interdependence was too marked and without any warnings. Thus, we decided to dedicate a couple of new paragraphs in the

revised version of the Introduction to better contextualize the approach followed. For the reviewer's convenience, we reported here below the new sentences:

*"In addition, the use of optical instruments provides simultaneously information about the interplay between structural connectivity and the emerging activity. This last feature is of extreme interest when we deal with high density (2D and 3D) neuronal assemblies. In 2017, Marom and colleagues developed an optical-accessible in vitro model of 3D networks by injecting a genetically encoded calcium indicator [18] to probe the variations of the emerging electrophysiological activity under their natural development. Many the works in the literature dealing with in vitro cultures exploit functional connectivity hints to infer structural properties [19]. Indeed, functional connectivity is strongly influenced by how it is measured (correlation- vs information-theory based algorithms for example), and some caveats are required to translate functional features into structural ones. First, functional measures are subject to fluctuations if achieved from short periods of recordings. Theoretical studies demonstrated the requirement of at least 10 minutes of recordings to avoid unstable results [20]. In addition, the achieved functional properties of a network can be considered predictors of the structural ones, only at small-scale, i.e., considering small ensembles of neurons [21]. Finally, it is worth mentioning the state-dependency of the functional connectivity: in other words, the same structural connectivity can originate different functional networks which exhibit different topological features. Thus, taking into consideration the weak points of the approach, the correlation between structural and- functional connectivity can be considered is indeed a good indication toon infer topological properties reciprocally, but taking into considerations the weak points of the approach. In this work, although knowing the weak points of the functional-structural relationships, we implemented precise structural connectivity rules in the computational model, driven by the inferred functional networks."*

11. Line 248

what is the rationale of scale free only in the readout layer? Layer 1 is scale free and others no? you do not present any real rationale for it, although I guess this is based on less physical constraints on bottom layer which is seeded separately. As I said this kind of information which related your model to the experimental model you try to reproduce, has to be presented at the beginning of the results and not just in methods

The reviewer's observation is extremely relevant. The rationale to model with a scale-free topology only one layer (the bottom one) is driven by the fact that we are able "to read" from a functional point of view (using MEA devices) only the bottom one. In vitro experimental studies showed the possibility to a network organization with a scale-free connectivity (Eythan Marom; Bonifazi, Science). As suggested, in the revised version of the manuscript, we added in the Results subsection describing this configuration, a short recap of the experimental/modelling conditions. For the reviewer's convenience, we reported here below the one related to this configuration.

*"From a functional point of view, different types of connectivity features were observed in 2D in vitro models [19,28–30]. Among them, a scale-free (SF) distribution of the functional connectivity was observed both in hippocampal [31] and cortical [20] circuits, indicating the presence of hubs which are suggested to coordinate the activity of the entire circuitry under examination [19,31]. As a maintenance of the topological features between functional and structural networks can be kept [22], we reproduced the functional features of the SF network in the 3D model (details on the implementation can be found as Rule 2: SF connectivity). In particular, we established a SF connectivity first in each layer of the 3D network (Figure 7, $SF_{all}^{3D}$), then only in the readout or bottom layer (Figure 7, $SF_{L0}^{3D}$). In this case, the connectivity within the other layers was maintained as in the previous case (i.e., the reference connectivity rule where the connections were established following a Gaussian probability (details are reported in Rule 1: Gaussian probability). The connections between the layers were not modified (i.e., the reference connectivity following Gaussian probability). The 2D reference for these 3D networks was a 2D SF network (Figure 7, $SF^{2D}$). The results of these*

*configurations were compared against the ones obtained before with the reference connectivity for both 2D and 3D (Figure 2, Figure 7 $G^{2D}$ and $G^{3D}$)."*

12. <u>Figure 6</u>
    while only MFR and MBR are plotted here? before you plotted more metrics. it seems only for MFR in RND and MC shows some good match. in MBR there is no real good match. also specify if the bar plots represent the 25-75% percentiles of your model so it can be compared to the 25-75% percentile of the experimental data. Although it could be obvious this has to be clearly stated
    As reported in the discussion, our model suffers in the fitting of other statistics like the duration of the bursts and network bursts that result to be longer than the experimental ones, especially because the metrics are averaged on both the excitatory and inhibitory populations (this choice was made because *in vitro* we cannot really distinguish between the two when evaluating the dynamical parameters). However, the inhibitory cells display particularly long bursts in our model, therefore they raise the overall values for these parameters, especially when considering the choral activity of the network. Therefore, our choice was to insert only the more meaningful metrics in our opinion.
    Regarding the mentioned metrics, it is indeed true that the $G_{rnd}^{3D}$ and the $G_{5C}^{3D}$ show a better "performance" with respect to the other configurations, and in some way that is maintained for the MBR values, although less obvious.
    Concerning the representation of the percentiles, the explanation of the box plots is present in the materials and methods (moved to a dedicated section Statistical Analysis in the revised manuscript), and following the reviewer's suggestion, we reported the meaning also in the caption of Figure 6, as reported below:

    *"... (**A**) Mean firing rate, (**B**) mean-bursting rate, and (**C**) burst duration of the different configurations. The box indicates the 25–75 percentile of the simulated data. The dashed black lines in the panel (**A**) and (**B**) indicate the 25-75 percentiles of the experimental data distributions..."*

13. <u>Line 489-492</u>
    where are 3 configurations? one i guess is the non-restricted one but here you discuss two.
    We apologize for the confusion, the configurations with clusters of neurons projecting connections to the other layers are two (the ones discussed afterwards, i.e., the C and MC), that are to be added to the random (RND) and the reference (FREE) configurations discussed before that. The manuscript was revised to correct the mistake as below:

    *"Two different types of clusters were examined: in the first case (Fig 6G), the inter-layer connections were originated from neurons positioned in a 278-µm radius from the center of the culture itself ( $G_{C}^{3D}$); in the second (Fig 6H), the source neurons were located in 129-µm radius circles centered in 5 different locations of the culture ( $G_{5C}^{3D}$). In all the new configurations, the number of source neurons for inter-layers connections results to be around 360, that is 33% of the total population of each layer."*

**Reviewer #3**

It is an interesting work which proposes a computational approach to explore different connectivity configurations able to reproduce the activity patterns recorded by MEAs in 3D cortical networks.
We thank the reviewer for his/her positive comment and for having captured the goal and the aims of the developed model.

1. Concerning the % of inhibitory neurons (optimal value 20%, Fig 1), aren't there experimental hints to pre-define the number or density of the 2 main neuron types (excitatory and inhibitory)? Furthermore, I guess the connectivity impacts a lot on the firing patterns; it is not clear how they are connected (excitatory to inhibitory, auto-inhibition, inhibitory to excitatory…)
   The connectivity between the different types of cells is essential to keep the right balance that ensures the onset of physiological complex dynamics. For this reason, we chose to create the following connections: excitatory to inhibitory, excitatory to excitatory, inhibitory to excitatory. This choice is driven by experimental findings that show that these kinds of connections emerge in *in vitro* dissociated networks. To clarify the concept in the MS, the following sentence was added (line 445, first submission manuscript):

   *"Only excitatory to excitatory, excitatory to inhibitory, and inhibitory to excitatory connections were implemented in the present work driven by experimental findings"*

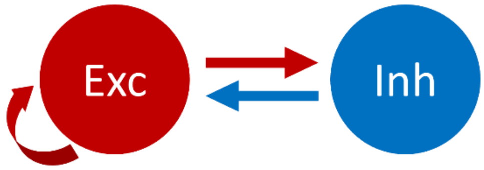


2. It is not clear then if all the different firing properties are averaged across all neurons (of the bottom layer), or only excitatory
   The metrics are averaged on both the excitatory and the inhibitory populations of the bottom layer. Following the reviewer's observation, we updated the manuscript as in the following (line 539, first submission manuscript):

   *"From the spike trains of both the excitatory and inhibitory populations of the readout layer, burst events…"*

3. Fig 2 E, which time bin?
   The bin size for the Figure 2E was 1 ms. We modified the caption of the figure specifying this value (line 195, first submission manuscript):

   *"(E) Average Spike Times Histogram (STH) of a 2D and a 3D exemplary configuration (bin = 1 ms)"*

4. It could be interesting to better comment the number of layers wrt biological properties of the cortical regions (e.g thickness)
   The thickness of the 3D networks (and consequently) the one of the 3D *in silico* model has been tailored to realize an engineered tissue able to:
   i) guarantee the survival of the neurons placed in the hidden layers since nutrients have to diffuse;
   ii) realize a structure with a thickness comparable with acute/organotypic slices;
   iii) define the minimum requirements (in terms of thickness) to observe changing in the dynamical properties of the networks with respect to the 2D counterpart.

5. Fig 3: the config with 3 layers (in E and F) seems to differ from some of the other configurations

We thank the reviewer for the observation. We indeed observe some differences, in particular the 3-layer configuration has a little more variability than the others. However, none of the considered comparisons proved to be statistical.

6. Fig 4 B. Slope: what does it mean? Linear fitting?
The degree distributions of the total connections were fitted with a power-law model. The slope refers to the linear fitting of the data in the log-log plot.

7. Fig 6: for the BD, isn't there any experimental reference value?
We thank the reviewer for the question. Indeed, we extracted the values for the BD from our cultures, however we did not plot them as they were partly outside the range of the plot. In fact, we are aware that this is a limitation of our model, that we will address in the future, possibly with the introduction of other parameters that are better able to modify the length of the bursts, especially of the inhibitory population. We stated this limitation in the discussion section, we report below the statement for the reviewer's benefit (line 370-373, first submission manuscript):

*"Despite the aforementioned limitations and tailored working hypotheses, eventually, the developed model is able to fit and mimic different features (MFR, MBR, percentage of inhibitory neurons) of 3D networks (and the relative differences). The lack of accuracy in the fitting of other statistics like the duration of the bursts and network bursts that result to be longer than the experimental ones, can partially be explained by the choice of the computational strategy (model) that does not provide any intrinsic description of the bursting activity in terms of ionic channels (e.g., persistent sodium channels) or by the absence of any form of facilitation/depression mechanisms (short term plasticity) at the level of the synaptic models."*

8. It is not well defined the difference between the configurations Free and Rnd ; also in rows 487-488 "random and unrestricted (Free).. and Random (rnd)" is confusing
We thank the reviewer for the observation. We greatly revised the manuscript to guide the reader through the configurations, by modifying the text and by adding Figure 7 in the Dataset section. For the reviewer convenience, we report the modified paragraph, the new Figure and table.

*"Assuming these hypotheses, we evaluated different dispositions of the neurons that generated the 3D connections. The underlying issue for this change was that it is not yet experimentally understood how the physical links are distributed in the z-direction in in vitro cultures. Therefore, the first, and reference, approach was random and unrestricted ($G^{3D}$). Then, we explored the effect of limiting the number of neurons that generated the 3D links ($G^{3D}_{rnd}$, Fig 6F). Afterwards, we investigated whether the connections were originated in specific regions of the network. Two different types of clusters were examined: in the first case (Fig 6G), the inter-layer connections were originated from neurons positioned in a 278-µm radius from the center of the culture itself ( $G^{3D}_{C}$); in the second (Fig 6H), the source neurons were located in 129-µm radius circles centered in 5 different locations of the culture ($G^{3D}_{5C}$). In all the new configurations, the number of source neurons for inter-layers connections results to be around 360, that is 33% of the total population of each layer."*

| | Description | # sim |
|---|---|---|
| $G^{2D}$ | Gaussian connectivity rule in a single layer | 20 |
| $G^{3D}$ | Gaussian connectivity rule within every layer of the 3D population. | 25 |
| $G^{3D}_{rnd}$ | Gaussian connectivity rule within every layer of the 3D population. The neurons that project to the other layers (source neurons) are a random subset of the total neurons of each layer (Figure 6F) | 4 |
| $G^{3D}_{C}$ | Gaussian connectivity rule within every layer of the 3D population. The source neurons are a located in a central cluster of each layer (Figure 6G) | 4 |
| $G^{3D}_{5C}$ | Gaussian connectivity rule within every layer of the 3D population. The source neurons are a located in five clusters (Figure 6H) | 4 |
| $SF^{2D}$ | Scale Free connectivity rule in a single layer | 28 |
| $SF^{3D}_{all}$ | Scale Free connectivity rule within every layer of the 3D population. Each neuron could project afferences to the other layers. | 22 |
| $SF^{3D}_{L0}$ | Scale Free connectivity rule within only the readout (bottom, L0) layer of the 3D population. The connections within the other layers follow the Gaussian connectivity rule. Each neuron could project afferences to the other layers. | 12 |

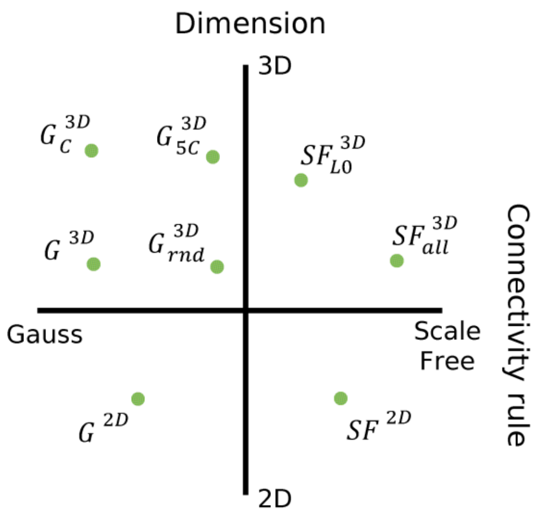


**Figure 7. Representation of the dataset.** All the configurations analyzed in the paper are reported here with their abbreviation and a brief description. For all the 3D, the connectivity between the layers follows a Gaussian distribution scaled over distance as reported in Sect. 3D connectivity.

9. It should be discussed a more detailed approach in modelling. If one uses multicompartmental neuron models in microcircuits, more "bottom-up" connectivity patterns could emerge (e.g. geometrical intersection among morphologies, anisotropy …); this could validate the connectivity properties emerged by the statistical rules tested here

   The reviewer captured a fundamental point in the genesis of the computational model. Detailed or reduced approaches for modeling the single neuron? Our choice was essentially driven by the experimental know-how. When dissociated cortical networks are extracted from rat embryos and plated over MEAs, we do not exactly know the kind of cortical cell, from which layers were extracted, etc. In addition, since *in vitro* cultures organize in dense networks, we cannot solve in detail their spatial organization too. The choice to use a compartmentalized model would be too hard to contextualize in our experimental framework since too many variables are "blind". A simpler point neuron model based on LIF dynamics, although introducing intrinsic limitations, allows to simulate with less unknown variables the dynamics of the entire network. In the revised version of the manuscript, we seriously took into account this comment by expanding in the Discussion Section the limitation of our computational approach. For the reviewer's convenience, we reported here below the new added section:

   *"In addition, it is worth noting that the rationale behind the genesis of the computational model is based on a reductionist approach: in our model, we choose to describe the dynamics of the single neuron by means of a mono-compartment model whose electrophysiological properties are described by means of LIF equations. The choice to neglect a multi-compartment approach with complex morphology, as well as biophysical models of the ionic channels, is driven by the lack of experimental evidence relative to the in vitro biological substrate. As already stated, 3D networks from dissociated cultures establish a dense connectivity and we are not able to solve with sufficient precision where synapses are placed (i.e., in which segments) or how the dendritic arborization develops among the layers. Finally, during the dissociation procedure (cf. Sect., In vitro model), we do not have total control on where the neurons are extracted from (i.e., from which cortical layers) and this limits the choice of the neuronal morphology. Thus, although it could theoretically be possible to implement a 3D model*

*with complex neuronal structures, the setting of its numerous parameters makes its applicability prohibitive for the goal of this work."*

10. Table 1: I_noise: is it synaptic noise? What is gl in Values column?
    We thank the reviewer for the question. $I\_noise$ was introduced to simulate the afferences to the neurons. It is necessary to keep the neurons in a responsive dynamical state to other potential synaptic stimuli, therefore it is a persistent fluctuation that constantly influences the neurons, it is not directly linked to synaptic events. The $g_l$ in the Values column is the Leakage conductance of the previous row.

11. Are the inhibitory and excitatory neurons modelled in the same way (same parameters)?
    Is the synaptic time constant the same for exc and inhib?
    How and c are set?
    In general, the parameter tuning is poorly explained and it seems no validation is provided
    We thank the reviewer for the questions. Firstly, the two types of neurons have the same parameters as listed in Table 1 of the Materials and Methods section, except for the synaptic parameters and equations. Indeed, we swept the space of the parameters, as for example different axonal velocities, but the change broke the balance between the two populations and did not produce the spiking and bursting dynamics. The time constant for the decay of the two currents is the same for the reason mentioned above. If either was changed, the network was thrown off its balance and its dynamics was disrupted. As for the third question, the parameter $c$ (Equation 3) was chosen as a multiplying factor in order to make the value dimensionally comparable to the voltage values. Finally, we totally agree with the reviewer's last observation. Indeed, in the revised version of the manuscript, we added the parameter tuning analysis as Supplementary Information in order to not interrupt the flow of the simulations to answer the scientific question at the basis of the developed computational model. In general, we either referred to values found on the literature, or we performed specific tests to calibrate the model. Below, we report and example of this empirical process on the Inhibitory synaptic weight. We swept different values of this parameter, and we plotted the different metrics obtained (we report below two of these, the MFR and MBR). Then from these, we identified the value that was likely to guarantee the most likelihood with the experimental (i.e., the one that allowed a physiological firing and bursting rates of both the excitatory and the inhibitory population without letting these values diverge).

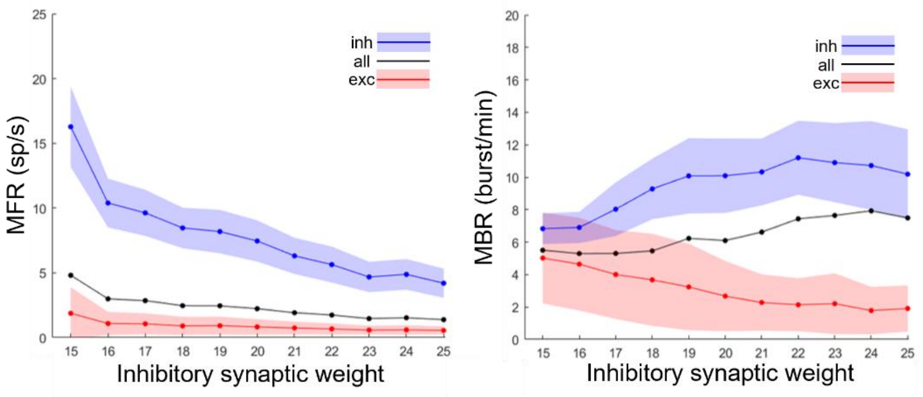


However, following the reviewer's suggestion we added a paragraph in the text (line 505, first submission manuscript) to better explain the tuning of the parameters, as follows:

*"The parameters used for the simulations were either extrapolated from the literature or calibrated with empirical trials, where the values of the parameters were chosen in order to guarantee a stable and physiological dynamics of the cells. A list of the swept parameters can be found in the supplementary information (Figure S5 and Table S1)."*

12. Rule 1: how the threshold (8%) is defined to implement the pruning?

We thank the reviewer for the question. We chose the percentage following an empirical process as before. In particular, we spanned the value from 5 to 11 % and identified the value that guaranteed a good excitatory overall firing avoiding the divergence of the inhibitory population. The reason for the chosen range is that outside the network either diverged or could be considered silent.

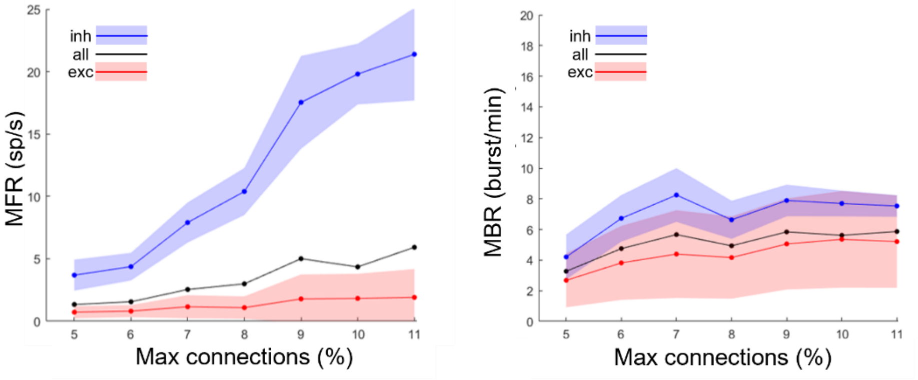


13. 3D connectivity models: the probabilistic control over distance seems to be isotropic. It should be discussed in limitations

Yes, it is. The implemented connectivity rule is isotropic. As suggested, we discussed such a choice in the revised version of the Discussion. For the reviewer's convenience, the following statement has been added:

*"To simulate these 3D environments, more complex connectivity rules should be taken into account: the working hypothesis of an isotropic connectivity fails and as consequence, the topographic displacement of the neurons in the 3D space and of their connections requires the implementation of an anisotropic connectivity induced by the irregularity of the scaffold."*

14. In general, a limitation paragraph on the model should be inserted

The revised version of the manuscript contains a new section collecting all the limits of the model. For the reviewer's convenience, we reported here below the new paragraph:

"***Model limitations***

*Indeed, our model was tailored to the peculiar experimental configuration of 3D network organized in ordered and engineered layers obtained thanks to glass microbeads. These experimental constraints made the design of the model easier as it neglected the irregular 3D positioning of neurons assumed when they grow in amorphous scaffolds like hydrogels [41]. To simulate these 3D environments, more complex connectivity rules should be taken into account: the working hypothesis of an isotropic connectivity fails and as consequence, the topographic displacement of the neurons in the 3D space and of their connections requires the implementation of an anisotropic connectivity induced by the irregularity of the scaffold.*

*In addition, it is worth noting that the rationale behind the genesis of the computational model is based on a reductionist approach: we choose to describe the dynamics of the single neuron by means of a mono-compartment model whose electrophysiological properties are described by means of LIF equations. The choice to neglect a multi-compartment approach with complex morphology, as well as biophysical models of the ionic channels, is driven by the lack of experimental evidence relative to the in vitro biological substrate. As already stated, 3D networks from dissociated cultures establish a dense connectivity and we are not able to solve with sufficient precision where synapses are placed (i.e., in which segments) or how the dendritic arborization develops among the layers. Finally, during the dissociation procedure (cf. Sect., In vitro model), we do not have total control on where the neurons are extracted from (i.e., from which cortical layers) and this limits the choice of the neuronal morphology. Thus, although it could theoretically be possible to implement a 3D model with complex*

*neuronal structures, the setting of its numerous parameters makes its applicability prohibitive for the goal of this work.*
*Despite the aforementioned limitations and tailored working hypotheses, eventually, the developed model is able to fit and mimic different features (MFR, MBR, percentage of inhibitory neurons) of 3D networks (and the relative differences). The lack of accuracy in the fitting of other statistics like the duration of the bursts and network bursts that result to be longer than the experimental ones, can partially be explained by the choice of the computational strategy (model) that does not provide any intrinsic description of the bursting activity in terms of ionic channels (e.g., persistent sodium channels) or by the absence of any form of facilitation/depression mechanisms (short term plasticity) at the level of the synaptic models."*

15. Some information about the propagation along layers (from in silico available data) could be added
    The reviewer is right, information about the propagation among the layers is indeed a very interesting feature to study, however it goes a little beyond the scope of the current work. In fact, our work focuses on reproducing the experimental data obtained with planar MEAs, which give no indication on the propagation between the layers. For this reason, we did not particularly focus on this parameter. However, we agree with the reviewer on the importance of the propagation, and in the revised version of the manuscript we included some considerations on the following parameters: the delays in the peak activation of the network bursts, obtained from the IFR traces (Figure 1, second row), the similarity values between the activation times of the network bursts of different layers (Supplementary information, Figure S2), and the involvement of the nodes in the population events (Supplementary information, Figure S2). Also, we added a paragraph discussing the propagation in the results, as follows:

    *"The result of the chosen combined parameters of these networks (Table 1) gave rise to a sustained spiking and bursting activity also in the 3D configuration, with network bursts that involved almost the entire network, as shown by the 60-s raster plots of a 4-layer network in Figure 1A-D (and the respective close-ups of a single event in the lower row). Considering the population events, we considered their propagation among the layers. Qualitatively, the IFR traces reported in Figure 1 (lower row) highlight the time stamp of the peak associated with one of the network bursts. From these values, we inferred the delay in the propagation of the event between the layers. In particular, this representative event started from the second layer, propagated to the third and the first ones and only then arrived to the fourth layer, that is the most active. In general, we observed a high variability in the generation and propagation of the network events (as demonstrated by the similarity maps of Fig S2 B-D, obtained with the Victor-Purpora statistics [27] described in the Supplementary Information File S1), possibly brought by the intricate connections established in the 3D space. Moreover, we observed that the number of involved neurons during these events significantly decreases if compared to 2D networks (Fig S2 A). This fact suggests that the stereotyped activity of the network is on some level severed by the introduction of the third dimension, as a lower percentage of cells is involved in the network event.*
    *However, considering the higher level of complexity in the evaluation of the whole network and the lack of an in vitro experimental counterpart (data from planar MEAs), the focus of our analysis was on the examination of the metrics obtained only from the bottom (or readout) layer of 3D networks and from 2D ones to try inferring some differences based solely on this reduced observation point of view."*

16. Typo row 284: where
    We thank the reviewer for the observation. The typo was corrected in the revised version of the manuscript.

17. I think in rows 490-494, it should refer to INTER-layer connections
    The reviewer is right. The sentence was modified from INTRA- to INTER-layer.

18. 10 seconds to settle the simulation; why so long?

    A settling time at the beginning of the was necessary to let the variables reach an equilibrium. The reason why it is 10 seconds long was because we tried and compared different single cell models before deciding for the LIF model. These other models had a more random and scattered spiking activity at the beginning of the simulations before the onset of the bursting network activity, which lasted up to 5/6 seconds. To even out the results among the different models, we chose to allow an arbitrary longer settling time (10 seconds) for all the simulations.

19. Row 549: “inter-burst event intervals maximum threshold (set at 100 ms)” not clear

    We change the sentence as follows:

    “the maximum interval that occurs between two consecutive events (set at 100 ms)”
